# Supplementary material for: Pinhole-seeded lateral epitaxy and exfoliation of GaSb films on graphene-terminated surfaces
Source: Nat Commun. 2022 Jul 18;13:4014. doi: 10.1038/s41467-022-31610-y (PMC9293962; doi:10.1038/s41467-022-31610-y)
Supplement: Supplementary file 1 — Supplementary Information [file 41467_2022_31610_MOESM1_ESM.pdf]

Supplementary Information:
Pinhole-seeded lateral epitaxy and exfoliation of GaSb films on graphene-terminated surfaces

Sebastian Manzo,[1] Patrick J. Strohbeen,[1] Zheng Hui Lim,[1] Vivek Saraswat,[1] Dongxue Du,[1] Shining Xu,[2] Nikhil Pokharel,[2] Luke J. Mawst,[2] Michael S. Arnold,[1] and Jason K. Kawasaki[1]

[1]*Materials Science and Engineering, University of Wisconsin-Madison, Madison, WI 53706*
[2]*Electrical and Computer Engineering, University of Wisconsin-Madison, Madison, WI 53706*

(Dated: June 2, 2022)

SUPPLEMENTARY FIGURES

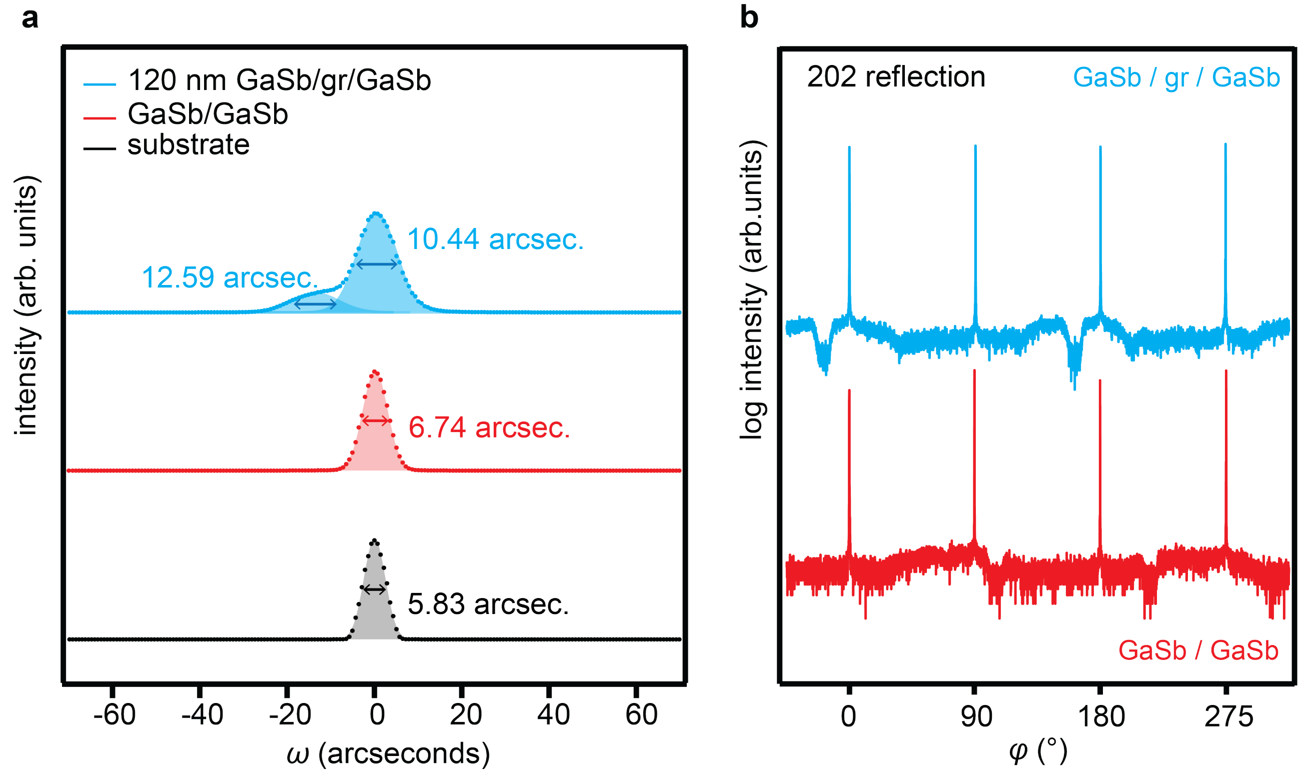

SUPPLEMENTARY FIGURE 1. **Extended x-ray diffraction of GaSb grown on graphene and on a bare GaSb substrate.** (a) Rocking curve of the 004 reflection for GaSb on graphene/GaSb (001), GaSb on GaSb (001), and a bare GaSb (001) substrate. (b) Pole figure ϕ scans of the 202 reflection, showing the expected 4-fold symmetry of an epitaxial GaSb film.

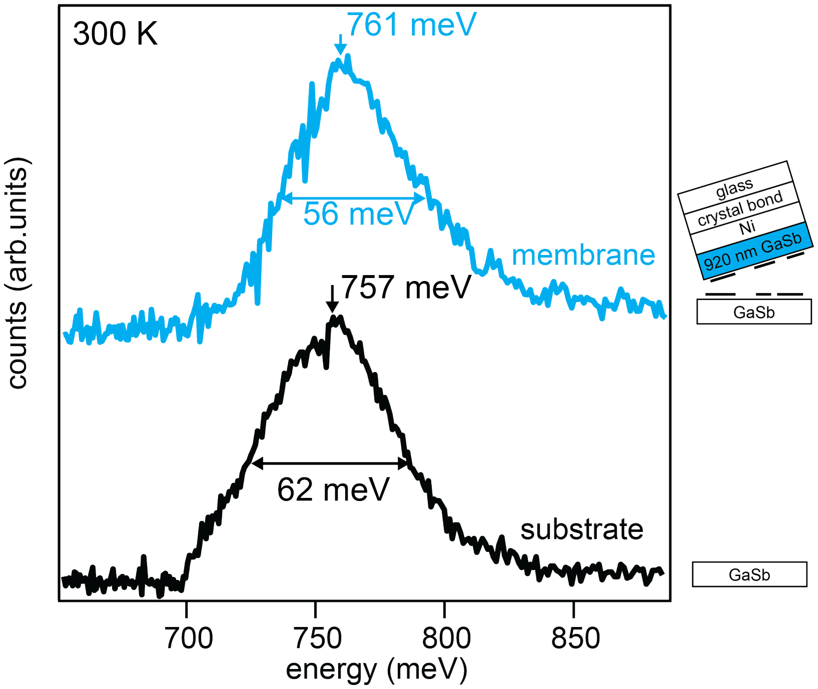

SUPPLEMENTARY FIGURE 2. **Normalized room temperature photoluminescence (PL) spectroscopy of a 920 nm thick exfoliated GaSb membrane (blue), compared to a bare GaSb (001) substrate (black).** The peaks at 761 meV (membrane) and 757 meV (substrate) correspond to the direct bandgap transition of GaSb. The membrane is slightly redshifted compared to a substrate, which we attribute to strain imparted by the Ni stressor layer. Both membrane and substrate peaks exhibit similar full width at half maximum of $\sim$ 60 meV.

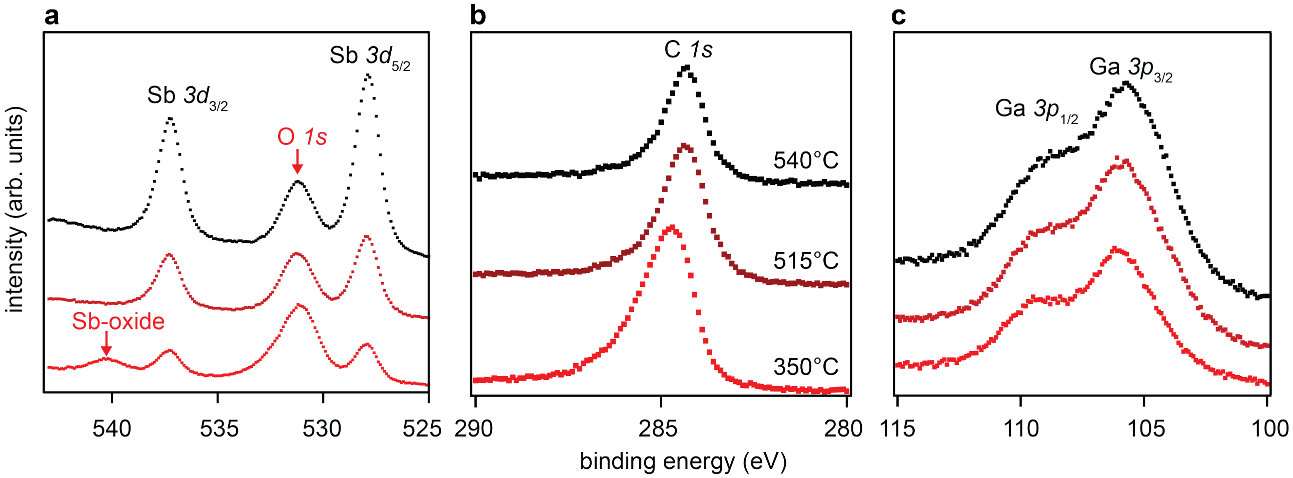

SUPPLEMENTARY FIGURE 3. **Extended in-situ photoemission spectra of graphene on GaSb (001) tracking the native oxide desorption as a function of annealing.** (a) Sb 3d and O 1s core levels. The Sb-oxide intensity goes to zero after the 515°C anneal, but residual atomic O 1s remains. (b) C 1s. After the 515°C anneal we observe a shift in the C 1s core level towards lower binding energy, which we attribute to de-oxidation. (c) Ga 3p. Ga-oxide components are not resolved in these spectra.

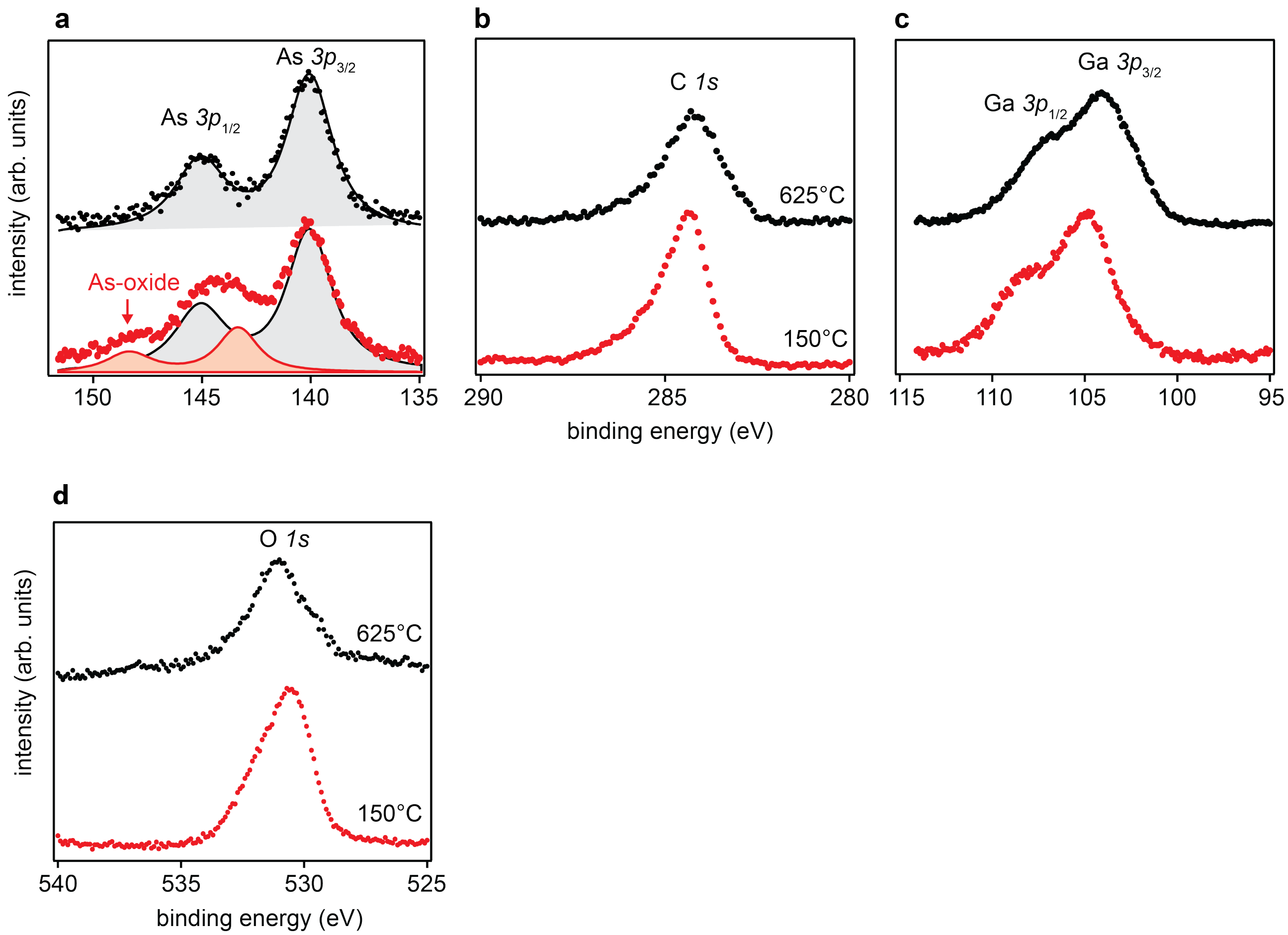

SUPPLEMENTARY FIGURE 4. **Native oxide desorption from a graphene/GaAs (001) sample.** The CVD-grown graphene was wet transferred to GaAs (001) after an HCl etch to remove some of the GaAs native oxides. (a) Phtoemission spectra of the As 3p core level. Arsenic (As) oxides are detected after the 150°C anneal (red curve) and are desorbed after the 625°C anneal (black curve). (b) There is a corresponding change in the C 1s lineshape. (c) Ga 3p core level. (d) O 1s core level.

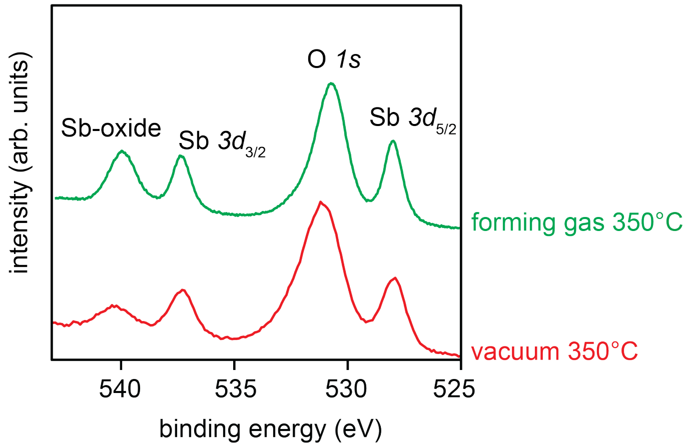

SUPPLEMENTARY FIGURE 5. **Forming gas annealing of graphene/GaSb(001).** Photoemission spectra of forming gas annealed graphene/GaSb (001) (green curve) compared to vacuum annealed graphene/GaSb (001) (red curve). Both spectra show significant Sb-oxides and O 1s. The forming gas annealed sample was annealed in a tube furnace at 350°C for two hours and then transferred through air to the XPS system. The vacuum annealed sample was annealed in ultrahigh vacuum in the XPS chamber, and thus did not get exposed to air after the anneal.

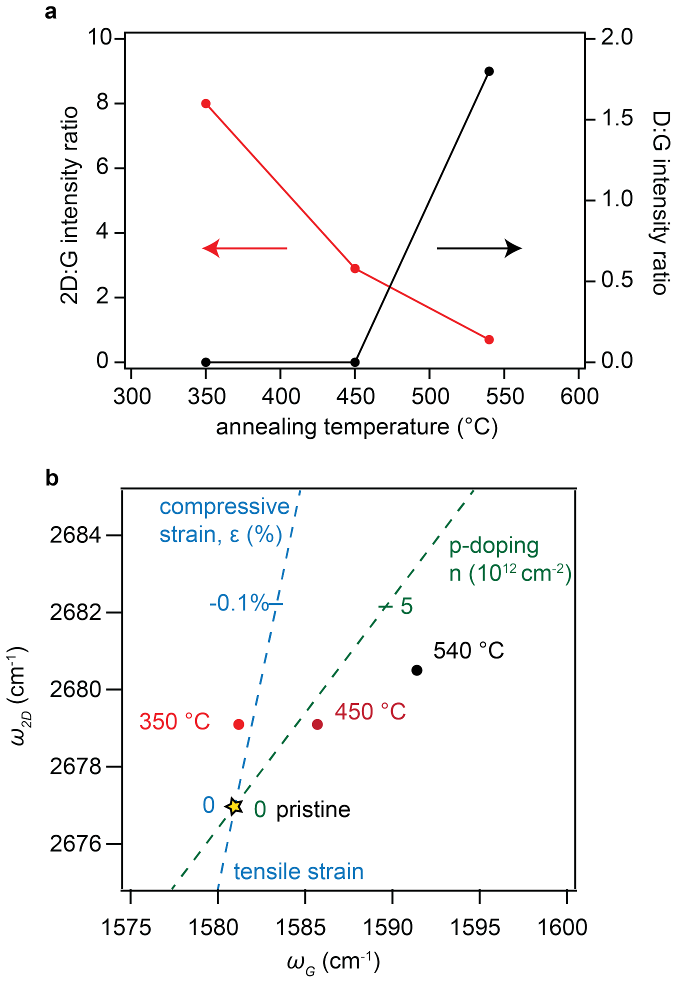

SUPPLEMENTARY FIGURE 6. **Extended Raman analysis of transferred graphene on GaSb (001), through the oxide desorption.** (a) $2D : G$ and $D : G$ integrated intensity ratios as a function of anneal temperature. Above 450°C, which was the onset of native oxide desorption from XPS and AFM measurements, the $D : G$ ratio increases (black curve), indicative of graphene defects. The $2D : G$ (red curve) decreases continuously across the range of anneal temperatures. (b) $2D$ versus G frequency, tracking changes in strain and doping in the graphene with increasing anneal temperature. The dependence of doping and strain on G and $2D$ frequency (dotted lines) is from Ref. [1].

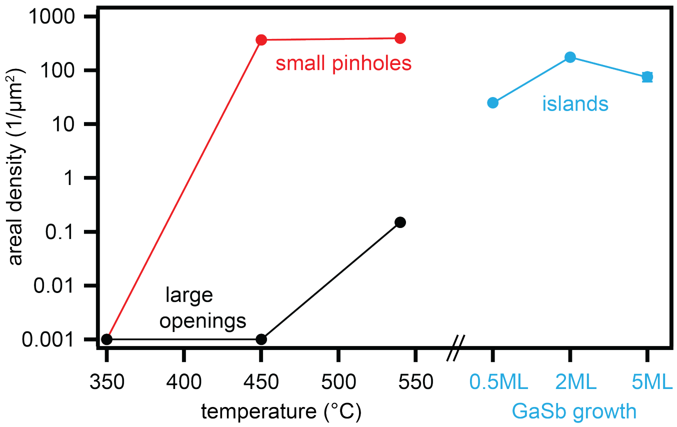

SUPPLEMENTARY FIGURE 7. **Areal density of pinholes and nucleated GaSb islands.** The density of small pinholes (diameter < 300 nm) was extracted from AFM measurements. The density of larger openings (diameter > 300 nm) was extracted from SEM. The strong correspondence between density of islands and density of pinholes is consistent with GaSb nucleation at the pinholes. From 2 ML to 5 ML of GaSb growth, the island density decreases due to the beginning of coalescence.

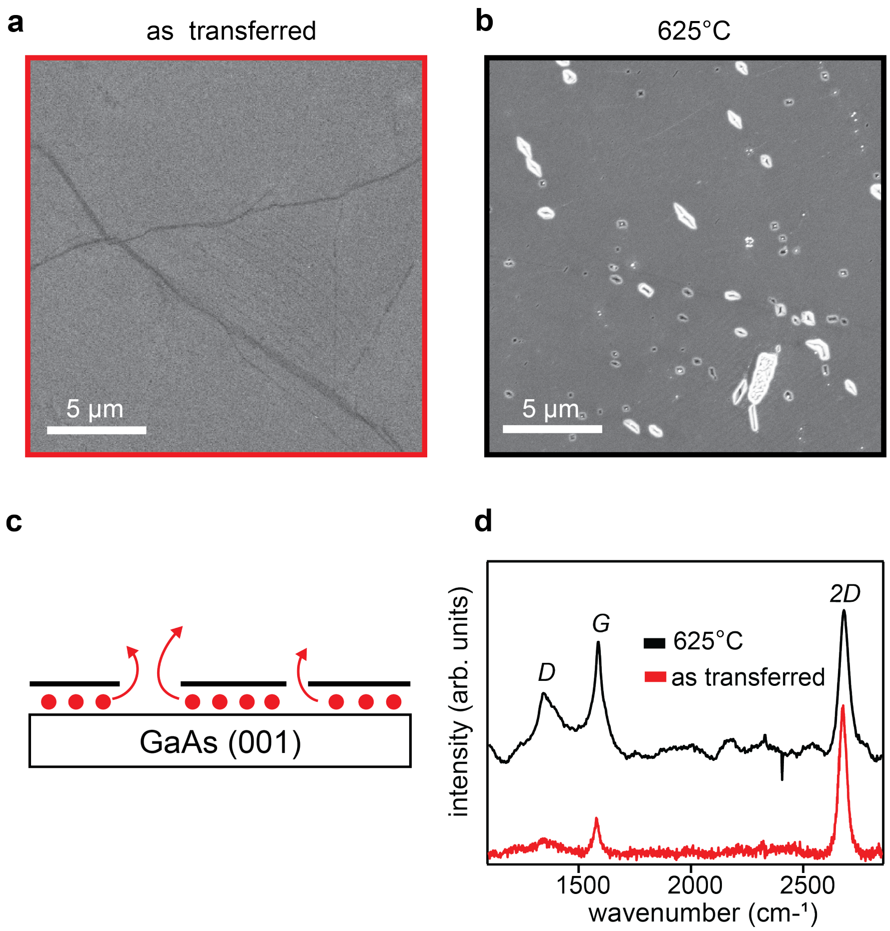

SUPPLEMENTARY FIGURE 8. **Pinholes formed in wet transferred graphene on GaAs (001).** (a) SEM image of graphene transferred to GaAs (001), before annealing. No pinholes are observed. The dark lines are wrinkles. (b) SEM image after annealing above the native oxide desorption temperature (625°C for 2 hours). Pinholes are observed. (c) Cartoon of the native oxide desorption. (d) Raman spectra before and after annealing above the native oxide desorption. We observed an increased Raman *D* peak after native oxide desorption (625°C).

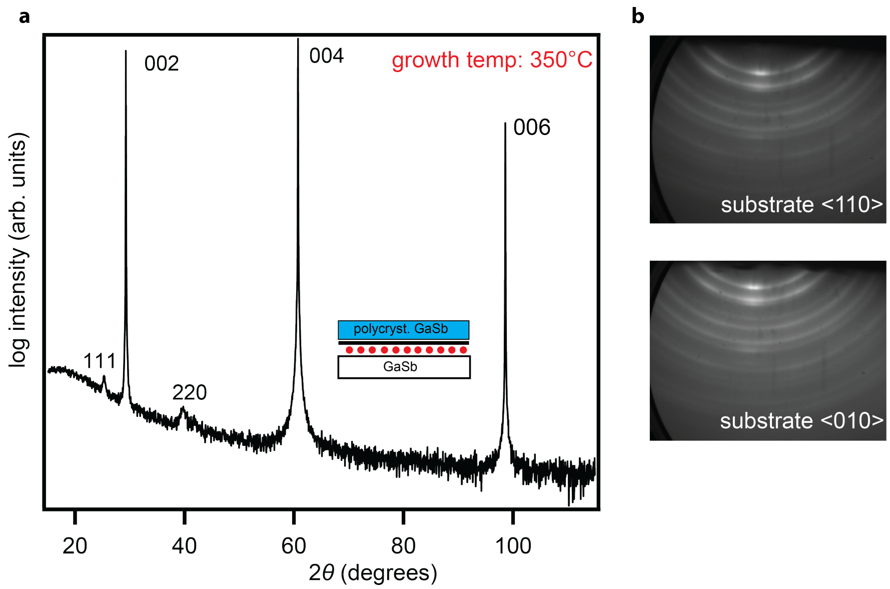

SUPPLEMENTARY FIGURE 9. **Electron and x-ray diffraction of GaSb grown on graphene-terminated GaSb below the native oxide desorption temperature.** (a) At a growth temperature of 350°C, holes in the graphene will not form, which means that pinhole-seeded epitaxy is not possible. As a result, a polycrystalline film is grown as evidenced by the presence of 111 and 220 reflections in the $\omega - 2\theta$ scan. (b) The rings present in the RHEED images of both <110> and <010> substrate orientations indicate that the film is polycrystalline.

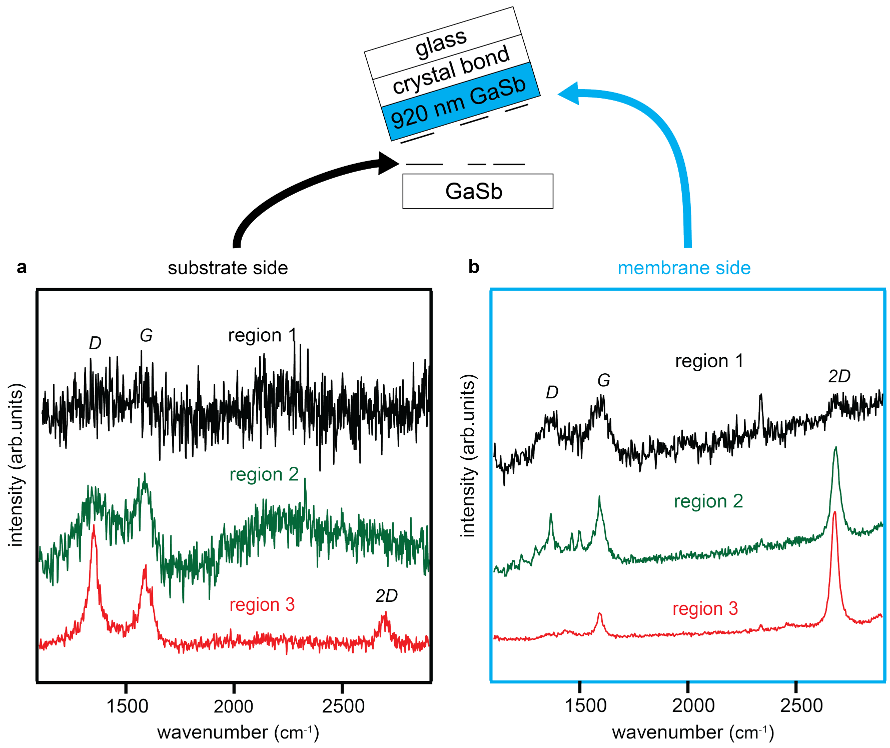

SUPPLEMENTARY FIGURE 10. **Raman spectroscopy of graphene on membrane and substrate post-exfoliation.** After exfoliation, graphene can be seen on both the exfoliated substrate and the membrane, indicating that some graphene has been torn away from the substrate. Additionally, the graphene quality is in-homogeneous on both sides, as evidenced by the variation in the D:G peak and 2D:G peak intensity ratios for the six representative regions being shown in (a) and (b).

SUPPLEMENTARY REFERENCES

[1] J. E. Lee, G. Ahn, J. Shim, Y. S. Lee, and S. Ryu, Nature communications **3**, 1024 (2012).
